# Malaria transmission and vector behaviour in a forested malaria focus in central Vietnam and the implications for vector control

**DOI:** 10.1186/1475-2875-9-373

**Published:** 2010-12-23

**Authors:** Wim Van Bortel, Ho Dinh Trung, Le Xuan Hoi, Nguyen Van Ham, Nguyen Van Chut, Nguyen Dinh Luu, Patricia Roelants, Leen Denis, Niko Speybroeck, Umberto D'Alessandro, Marc Coosemans

**Affiliations:** 1 Institute of Tropical Medicine Antwerp, Dept. Parasitology, Nationalestraat 155, B-2000 Antwerpen, Belgium; 2 National Institute of Malariology, Parasitology and Entomology, Dept. Entomology, Luong The Vinh street, B.C. 10.200 Tu Liem, Hanoi, Vietnam; 3 Centre for Malariology, Parasitology & Entomology of Ninh Thuan province, Vietnam; 4 Institute of Tropical Medicine, Dept. Animal Health, Nationalestraat 155, B-2000 Antwerpen, Belgium; 5 Institute of Health and Society, Université Catholique de Louvain, Brussels, Belgium; 6 Department of Biomedical Sciences, Faculty of Pharmaceutical, Veterinary and Biomedical Sciences, University of Antwerp, Universiteitsplein 1, B-2610 Antwerpen, Belgium

## Abstract

**Background:**

In Vietnam, malaria is becoming progressively restricted to specific foci where human and vector characteristics alter the known malaria epidemiology, urging for alternative or adapted control strategies. Long-lasting insecticidal hammocks (LLIH) were designed and introduced in Ninh Thuan province, south-central Vietnam, to control malaria in the specific context of forest malaria. An entomological study in this specific forested environment was conducted to assess the behavioural patterns of forest and village vectors and to assess the spatio-temporal risk factors of malaria transmission in the province.

**Methods:**

Five entomological surveys were conducted in three villages in Ma Noi commune and in five villages in Phuoc Binh commune in Ninh Thuan Province, south-central Vietnam. Collections were made inside the village, at the plot near the slash-and-burn fields in the forest and on the way to the forest. All collected mosquito species were subjected to enzyme-linked immunosorbent assay (ELISA) to detect *Plasmodium *in the head-thoracic portion of individual mosquitoes after morphological identification. Collection data were analysed by use of correspondence and multivariate analyses.

**Results:**

The mosquito density in the study area was low with on average 3.7 anopheline bites per man-night and 17.4 culicine bites per man-night. *Plasmodium-*infected mosquitoes were only found in the forest and on the way to the forest. Malaria transmission in the forested malaria foci was spread over the entire night, from dusk to dawn, but was most intense in the early evening as nine of the 13 *Plasmodium *positive bites occurred before 21H. The annual entomological inoculation rate of *Plasmodium falciparum *was 2.2 infective bites per person-year to which *Anopheles dirus s.s*. and *Anopheles minimus s.s*. contributed. The *Plasmodium vivax *annual entomological inoculation rate was 2.5 infective bites per person-year with *Anopheles sawadwongporni*, *Anopheles dirus s.s*. and *Anopheles pampanai *as vectors.

**Conclusion:**

The vector behaviour and spatio-temporal patterns of malaria transmission in Southeast Asia impose new challenges when changing objectives from control to elimination of malaria and make it necessary to focus not only on the known main vector species. Moreover, effective tools to prevent malaria transmission in the early evening and in the early morning, when the treated bed net cannot be used, need to be developed.

## Background

Long-lasting insecticidal materials are central in the prevention and control of malaria. To be successful they need to be adapted to the local epidemiological context. Vietnam has demonstrated that a substantial reduction of malaria-related mortality and morbidity is feasible. Political commitment to malaria control and internal and external funding were key to this success [1,2]. However, malaria elimination in Vietnam can only be achieved if the country is able to cope with the remaining malaria endemic foci. These foci are situated in the central highlands and along international borders of Laos and Cambodia where the majority of the severe cases and malaria-related deaths occurs [3]. These areas are remote, forested and populated by ethnic minorities living traditionally on forest related activities. These activities have been identified as a strong risk factor for malaria infection [4,5]. The vector present in this forested area, *Anopheles dirus*, is exophagic and exophilic, jeopardizing the impact of the traditional control measures [6]. To protect this specific human population, an adapted preventive measure, namely long-lasting insecticidal hammocks (LLIH), were proposed. LLIH were designed to control malaria in the specific forest context where people use hammocks. It consisted of a green nylon hammock to which Olyset^® ^netting was stitched. The netting was double the width of the hammock itself [7]. Half was sewn onto the back of the hammock while the other half served as a free flap to cover the person inside. A community based-cluster randomized intervention, assessing the impact of the LLIH, was implemented in the province of Ninh Thuan Vietnam and showed that LLIH reduced malaria incidence and prevalence, especially in the cluster with the highest malaria burden [8]. Knowledge of the behaviour of humans [9] and vectors are important for the success of this control measure. Therefore, an entomological study was conducted, in the framework of the above mentioned community-based trial, to assess the behavioural patterns of forest and village vectors and to assess the spatio-temporal risk factors of malaria transmission in this province.

## Methods

### Study place

The study was conducted in the province of Ninh Thuan in south-central Vietnam. The province has about 570,000 inhabitants and is divided into six districts. The Ra-glai people of Ninh Thuan combine living in villages along the road with a second home at their slash and burn fields in the forest. In the forest, houses are generally built on stilts whereas in the villages two types of houses, built on the ground or on stilts, are present. They depend on the forest for their subsistence where they are at high risk of getting malaria. They grow maize, cashew nuts, rice, beans and manioc, cultivate cash crops such as coffee and cotton, and exploit forest products, such as bamboo, resin, and hunting. The dry season in this province lasts from January to April, the rainy season from May till December. The mean annual rainfall is 725 mm per year with the mean temperature ranging from 25 to 30°C and the humidity between 70 and 80%. Malaria transmission shows a peak in May-June at the start of the rainy season and in October-November at the end of the rainy season.

### Mosquito collections

Five entomological surveys (November 2004, October and November 2005, October and November 2006) were conducted in three villages in Ma Noi commune and in five villages in Phuoc Binh commune. Collections were made in two different places per study village i.e. inside the village (subsequently called village) and at the plot near the slash and burn fields in the forest (subsequently called forest). In one village per commune collections were made in three different places on a road frequented by the inhabitants to go from the village to the forest plot (subsequently called 'way'). In each collection place, outdoor human landing collections were made during eight nights per survey. In the village, two houses were selected, each with one collector outdoors, in the forest two places were selected with each time two collectors, and on the 'way' each site was sampled by two collectors. From survey 3, indoor human collections were made inside the village in two houses by one collector to assess the indoor and outdoor mosquito density in the village. In the village and forest sites, collections were made from 18H till 06H by two teams. A first team worked from 18H to 24H and a second team from 24H to 6H. On the way from village to forest collections were made from 17H till 20H and from 04H till 07H.

Mosquitoes were stored by collection hour and morphologically identified in the field by use of a standardized key for medically important anophelines of Southeast Asia [10]. Mosquitoes were individually stored in small tubes over silica gel for subsequent analyses.

### Laboratory analyses

All collected mosquito species were subjected to enzyme-linked immunosorbent assay (ELISA) to detect *Plasmodium falciparum*, *Plasmodium vivax 210 *and *P. vivax 247 *circumsporozoite protein (CSP) in the head-thoracic portion of individual mosquitoes [11-13]. Positive CSP ELISA mosquitoes were confirmed by PCR. DNA was extracted from the ELISA homogenate using the QIAamp DNA micro kit (Qiagen) following the manufacturer's instructions. Amplification of *Plasmodium *was done by use of the primers PL1473F18 and PL1679R18 targeting the 18SrRNA [14], amplicons were subsequently cloned using the Original TA cloning kit according the manufacturer's instructions (Invitrogen, Carlsbad, California) and sequenced (GenoScreen, Lille, France). Sequences were used to confirm the *Plasmodium *species identification based on ELISA.

The morphological identification of the mosquitoes found positive for ELISA was confirmed by PCR using the PCR-RFLP for *Anopheles minimus *complex and *Anopheles pampanai *[15], and the allele specific PCR for *An. dirus complex *[16]. The identification of *Anopheles maculatus s.l*. was confirmed by sequencing (GenoScreen, Lille, France) the ITS2 rDNA region using primers ITS2A and ITS2B described in Beebe & Saul [17]. The sequences were blasted and compared with reference sequences described in Walton *et al *[18].

### Statistical analysis

The association between the *Anopheles *species with collection places (communes and forest/village combined) was evaluated by applying correspondence analysis (CA) on the contingency table of the collection data. CA is a multivariate technique most similar to factor analysis, but applicable on a two-way contingency table of observed frequencies. Chi-square distances provide a standardized measure of association between the rows and columns of the contingency table. CA transforms these association measures into metric distance measure and creates orthogonal dimensions upon which the categories can be placed to best account for the strength of association represented by the chi-square distances [19]. The degree of association or correspondence is reflected by the metric distances in the ordination plot, so that the smaller the distances between points, the "stronger" their association [19]. CA was performed using Stata 10.0 (Stata Corp. College Station, TX).

Differences in *Anopheles *densities (bites per man-night) were tested by a negative binomial regression taking into account the sampling design in which survey and village name were the clustering variables (command survey set in Stata). Collection place (village and forest), commune (Ma Noi, Phuoc Binh) and intervention were the explanatory variables. The study was not designed to assess the impact of the intervention on the mosquito density. Yet the LLIH intervention started in November 2004 and to account for the possible effect of intervention on the density it was included as explanatory variable in the analysis.

### Ethical consideration

The study protocol was approved by the ethical committees of NIMPE, Hanoi, Vietnam and of the Institute of Tropical Medicine, Antwerp, Belgium as well as by the Vietnamese Ministry of Health. The mosquito collectors and householders were informed about the objectives, process and procedures of the study and oral informed consent was sought from them. Collector candidates were invited among the adult village population and if individuals wished to withdraw, they were allowed to do so at any time without prejudice. Access to malaria diagnosis and treatment was guaranteed throughout the study.

## Results

### The spatial contact with the Anopheles species

**Figure 1:**
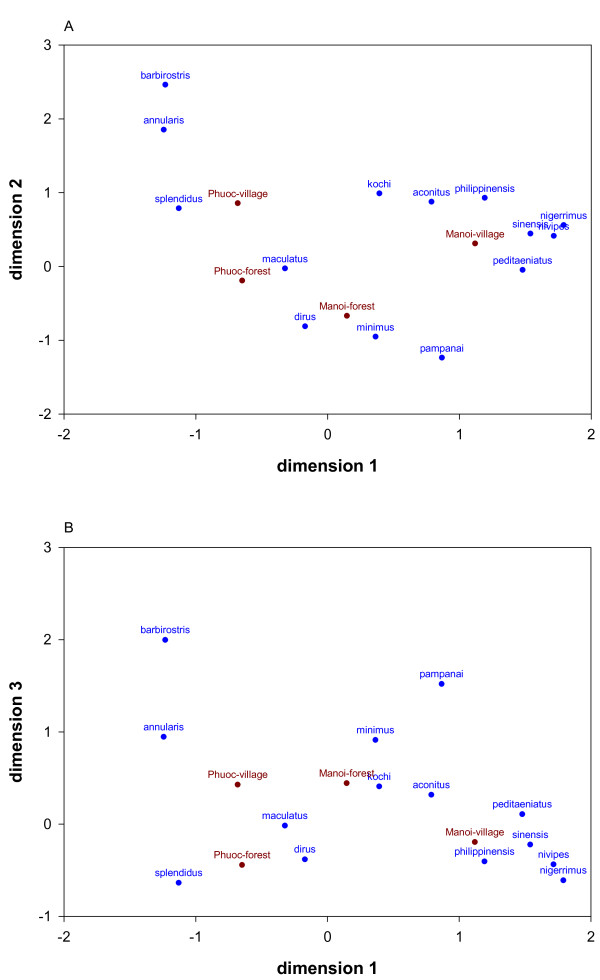
**Two-dimensional ordinations (plots A and B) of the 3-dimensional correspondence analysis showing the collected *Anopheles *species (i.e. raw categories, blue) and the village-collection sites (i.e. column categories, red)**. The dimensions in the ordination plot show the metric distances. The smaller the distances between points, the "stronger" their associate i.e. points of a certain category positioned close to each other are similar with regard to the pattern of relative frequencies across the other category. Village-collection sites; Phuoc-village: village in Phuoc Binh, Phuoc-forest: forest in Phuoc Binh, Manoi-village: village in Ma Noi, Manoi-forest: forest in Ma Noi.

In total, 24 different *Anopheles *species were collected outdoors in the village and forest. These collections counted 1821 person-nights divided over five surveys. Survey one counted 288 person-nights, surveys two, three and five 383 person-nights and survey four 384 person nights. During the first survey sampling was restricted to three villages. Following species were only sporadically collected i.e. less than 10 specimens for each of these species were found during the entire study: *Anopheles annandalei, Anopheles argyropus, Anopheles barbumbrosus, Anopheles crawfordi, Anopheles jamesii, Anopheles jeyporiensis, Anopheles monstrosus, Anopheles tessellatus, Anopheles vagus *and *Anopheles varuna*. These mosquitoes were not further considered in the analysis of the biting rates. The 14 remaining species were collected in both the forest and the village sites though at different densities resulting in a significant association between species, collection place and communes (Figure 1, Table 1). *Anopheles dirus s.s*., the only species of the complex occurring in this area [20], and *An. maculatus s.l*. were significantly associated with the forest in both Phuoc Binh and Ma Noi, while *An. minimus s.l*. was identified as a forest species associated with Ma Noi commune (Figure 1, Table 1). *Anopheles pampanai *was only collected in the commune of Ma Noi and showed an association with the forest (Figure 1). This trend was also observed in the regression analysis though the difference in density between forest and village was not significant for this species (Table 1). *Anopheles aconitus, Anopheles annularis, Anopheles nigerrimus, Anopheles philippinensis *and *Anopheles sinensis *were significantly more abundant in the villages. The village sites in Phuoc Binh were more similar in terms of species composition to the forest places of Ma Noi and Phuoc Binh than to the village sites in Ma Noi (Figure 1).

**Table 1:** The mean density (bites per man-night) per Anopheles species by collection place (forest/village), Commune (Ma Noi/Phuoc Binh) and intervention. The significance levels were obtained from a multivariate binomial regression analysis including collection place, communes and intervention as explanatory variables.

	Effect Forest/village				Effect Commune				Effect intervention			
			
*Anopheles* species	Forest		Village	p-value*	Ma Noi		Phuoc Binh	p-value*	Control		Inter-vention	p-value*
*aconitus*	0,059	<	**0,178**	0,013	0,157		0,061	0,143	**0,143**	>	0,038	0,005
*annularis*	0,022	<	**0,041**	0,003	0	<	**0,047**	0,000	0,000	<	**0,068**	0,000
*barbirostris*	0,012		0,056	0,133	0,001		0,043	0,061	0,031		0,020	0,434
*dirus*	**0,539**	>	0,106	0,011	0,364		0,415	0,972	0,355		0,449	0,632
*kochi*	0,009		0,020	0,126	0,013		0,013	0,846	0,009		0,018	0,141
*maculatus*	**2,941**	>	1,356	0,005	1,831		2,795	0,444	2,624		2,123	0,428
*minimus*	**0,240**	>	0,092	0,001	**0,332**	>	0,098	0,021	0,234		0,131	0,743
*nigerrimus*	0,030	<	**0,140**	0,020	**0,169**	>	0	0,000	**0,114**	>	0,003	0,011
*nivipes*	0,013		0,035	0,052	**0,051**	>	0	0,000	**0,035**	>	0	0,000
*pampanai*	0,026		0,012	0,125	**0,054**	>	0	0,000	0,022		0,021	0,548
*peditaeniatus*	0,122		0,172	0,511	**0,35**	>	0	0,000	0,173		0,090	0,837
*philippinensis*	0,003	<	**0,013**	0,045	0,011		0,004	0,348	0,009		0,004	0,667
*sinensis*	0,040	<	**0,102**	0,000	**0,141**	>	0,007	0,005	**0,098**	>	0,008	0,046
*splendidus*	0,229		0,141	0,000	0,007	<	**0,326**	0,000	**0,294**	>	0,007	0,026

* No adjustments were made to account for multiple testing. These corrections have been challenged [30] and the use of uncorrected p-values was preferred as indicative of an effect.

Overall the species density in the study area was low with on average 3.7 anopheline bites per man-night and 17.4 culicine bites per man-night (BMN). The mean outdoor *Anopheles *density in the forest was 4.3 BMN and in the village 2.5 BMN (p = 0.007). In the village the *Anopheles *outdoor biting density (2.5 BMN) was significantly higher than the indoor biting density (0.7 BMN) (p = 0.004). A similar pattern in the village was observed for *An. dirus *(0.12 BMN outdoors versus 0.02 BMN indoors, p = 0.035), *An. maculatus s.l*. (1.47 BMN outdoors versus 0.42 BMN indoors, p < 0.01) *and An. minimus s.l*. (0.07 BMN outdoors versus 0.008 BMN indoors, p = 0.023).

### The temporal contact with the Anopheles species

**Figure 2:**
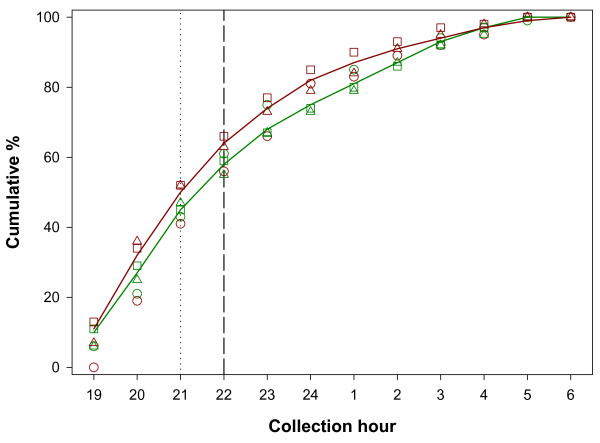
**Cumulative biting rate of *Anopheles *species (straight line), *Anopheles dirus *(circles), *Anopheles maculatus s.l*. (triangles) and *Anopheles minimus s.l*. (squares) during the night. Green: collections in the forest, Red: collections in the village**. The vertical dotted line indicates the human sleeping time in the forest. The vertical dashed line indicates the human sleeping time in the village [9].

**Figure 3:**
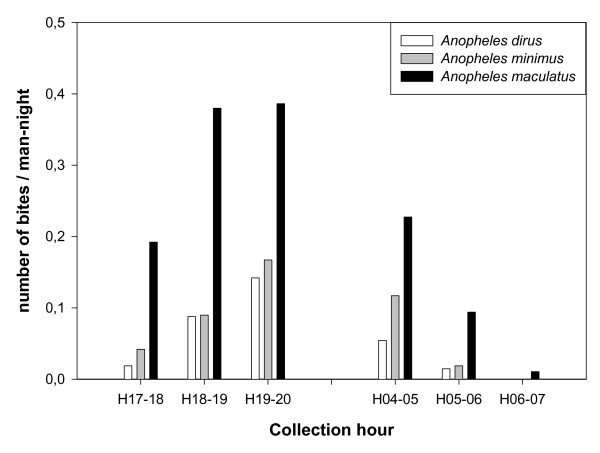
**The biting activity of *Anopheles dirus, Anopheles maculatus s.l*. and *Anopheles minimus s.l*. on the 'way' between village and forest plot (all data pooled)**.

The *Anopheles *species in the study area were early biters and almost no difference in biting time was observed between the forest and the village. In the forest, people were exposed to 45% of the total number of *Anopheles *bites by 21H which is the normal sleeping time for Ra-glai in the forest setting. In the village, where people go to bed no later than 22H, they were exposed to 64% of the *Anopheles *bites before going to sleep. The temporal exposure to the vector species, *An. dirus, An. maculatus s.l *and *An. minimus s.l*. was similar (Figure 2). These species were already active early in the night with up to 13% of the bites for *An. maculatus s.l*. by 19H. Likewise, this early activity was observed on the 'way' where the three species were collected before 18H. At the early morning *An. maculatus s.l. s*howed still some activity until 7H (Figure 3).

### Malaria transmission and the annual entomological inoculation rate

**Figure 4:**
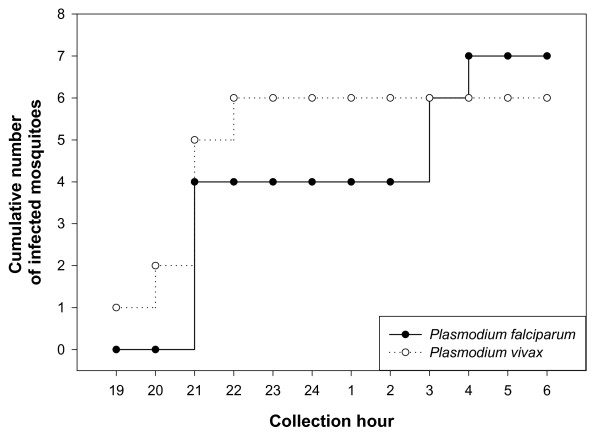
**Cumulative number of infected *Anopheles *mosquitoes observed during the night in the forest (all data pooled)**.

*Plasmodium *infected mosquitoes were only found in the forest and on the 'way' (Table 2) in both communes Ma Noi and Phuoc Binh. *Anopheles dirus s.s*. and *An. minimus s.s*. were found infected with *Plasmodium falciparum *while *Plasmodium vivax *was detected in *An. dirus s.s., An. maculatus s.l*. (molecularly identified as *Anopheles sawadwongporni*), and *An. pampanai*. The risk of people to contact infected mosquitoes in their forest plot was high during their evening activities as nine of the 13 infected mosquitoes (*P. falciparum *and *P. vivax *combined) were found by 21 hours (Figure 4). *Plasmodium falciparum *infected mosquitoes were also found in the forest in the second half of the night and on the 'way' two infective mosquitoes were observed in the early morning between 4-5 hours. The annual entomological inoculation rate of *P. falciparum *was 2.2 infective bites per person-year to which *An. dirus s.s*. contributed 85% and *An. minimus s.s*. 15%. The *P. vivax *annual entomological inoculation rate was 2.5 infective bites per person-year to which *An. maculatus s.l*. (molecularly identified as *An. sawadwongporni*) added 47%, *An. dirus s.s*. 37% and *An. pampanai *16%.

**Table 2:** Overview of the Plasmodium infected mosquitoes as detected by ELISA and confirmed by PCR.

		Number of *Plasmodium *sporozoite infected mosquitoes (number tested)		
		
Collection place	*Anopheles *species (morphological identification)	*P. falciparum*	*P. vivax 210*	*P. vivax 247*
Forest	*An. dirus*^1^	6 (642)	2 (642)	2 (485)
	*An. maculatus s.l*.^2^	0 (3454)	0 (3454)	2 (1826)
	*An. minimus s.l*.^3^	1 (264)	0 (264)	0 (166)
	*An. pampanai *^4^	0 (23)	0 (23)	1 (23)
				
Way	*An. dirus*^1^	2 (151)	0 (151)	0 (103)
	*An. maculatus s.l*.	0 (618)	0 (618)	0 (524)
	*An. minimus s.l*.	0 (204)	0 (204)	0 (144)
	*An. pampanai*	0 (37)	0 (37)	0 (37)
				
Village	*An. dirus s.l*.	0 (71)	0 (71)	0 (43)
	*An. maculatus s.l*.	0 (1009)	0 (1009)	0 (650)
	*An. minimus s.l*.	0 (62)	0 (62)	0 (41)
	*An. pampanai*	0 (5)	0 (5)	0 (5)

^1^All ELISA positive specimens were molecular identified as *An. dirus s.s*.

^2^All ELISA positive specimens were molecular identified as *An. sawadwongporni*

^3^The ELISA positive specimen was molecular identified as *An. minimus s.s*.

^4^The identification of the ELISA positive specimen was molecular confirmed as *An. pampanai*

## Discussion

In Ninh Thuan province, south central Vietnam, the biting nuisance of mosquitoes (Culicidae) in general and of *Anopheles *in particular was low both in the villages and in the forest, though the *Anopheles *density in the forest was significantly higher than in the village. All the *Anopheles *species considered in the analyses were found in both forest and village. Nevertheless, *An dirus, An. maculatus s.l*. and *An. minimus s.l*. were more abundant in the forest and had an early biting activity, corroborating previous observations [6].This study showed that these three mosquito species started their biting activity before 18H and that in the early morning *An. maculatus s.l*. was still active on the 'way'. Malaria transmission could only be detected in the forest and on the 'way'. The highest risk of contact with infected mosquitoes was during their evening activities. However, transmission also took place during the night and the early morning showing the importance to protect against mosquito bites from dusk to dawn. The annual entomological inoculation rate showed a level that was much lower than the one observed in Africa [21-23], but similar to what could be expected in malaria endemic foci in Southeast Asian mainland [24].

The Ra-glai people of Ninh Thuan combine living in villages along the road with a second home at their slash and burn fields in the forest. In these communities bed net (ITN) use in the villages was 85% but only 53% in the forest. About 20% slept unprotected in both places [9]. LLIH were introduced as a complementary control measure to protect these people against malaria transmission in the forest. The LLIH, utilized both indoors and outdoors, were more used during the day in both the villages and the forest (69% and 73% respectively), in the evening their use decreased to 54% in the villages and to only 21% at the forest plot, and at night they were hardly used (Peeters Grietens personal comment). Despite the low use of the LLIH during the night, the community based-cluster randomized trial showed an impact, mainly in the high risk group [8]. In Vietnam the risk of being bitten by a *Plasmodium *infectious mosquito is, as shown by this study, mainly during the early evening. Intervention, such as LLIH, that protects people during this period, when treated bed nets are not used, should be beneficial. Indeed, in Cambodia it has been shown that persons sitting in a LLIH are less bitten than persons sitting in a control hammock, though the personal protective effect of LLIH was variable according to mosquito species, villages and surveys [7]. The current study, suggested that no overall impact existed on the *Anopheles *density in forest, an expected result since the LLIH coverage was likely too low and too scattered to have any impact. Even if LLIH are not inducing full protection against the bites of all vectors, they may contribute protecting people. Insecticide-treated nets are however still needed as mosquitoes were active during the entire night and transmission also occurs during that period. Moreover, people are also exposed to mosquito bites when going to and from the forest plot hut in the early morning or in the evening. Elimination of malaria in this setting has to take this complexity into account and must rely on different control measures and personal protection methods.

*Plasmodium *infected mosquitoes were only detected in the forest and on the way from the village to the forest. This does not imply that malaria transmission is absent in the villages [5], but that the level of transmission was below the detection limits of the applied screening technique. As vector density was low inside the villages, fewer mosquitoes were assayed, decreasing the chance of sporozoite detection. Moreover, in areas of low transmission other techniques than estimating the entomological inoculation rate are needed to more accurately assess the trends in transmission. This can be done by serological test measuring the force of infection [25]. However, the CSP-ELISA technique followed by a PCR confirmation is still needed to identify *Anopheles *species bearing *Plasmodium *sporozoites. In Ninh Thuan province 24 different *Anopheles *species were identified among which four were incriminated as vectors of malaria, namely *An. dirus s.s*., *An. sawadwogporni*, *An. minimus s.s*. and *An. pampanai*. The contribution of the different *Anopheles *species to the transmission of the two *Plasmodium *species seemed to be different, though the numbers were too low to support this statistically. *Anopheles dirus *was the main vector for *P. falciparum *whereas *An. sawadwongporni *played an important role as *P. vivax *vector. In areas where *P. falciparum *and *P. vivax *co-occur, successful control leads often to a shift from *P. falciparum *to *P. vivax *[26-28]. The elimination of *P*. *falciparum *may even reveal *P. vivax*, previously hidden in mixed infections [29]. As the extrinsic incubation period of *P. vivax *is shorter, other *Anopheles *species, often considered as secondary vectors, may become important in malaria transmission in Ninh Thuan Province. This may change the epidemiology of the disease and make vector control more complex. *Anopheles sawadwongporni *for example showed to be a very early biter and was still active after 6 am. It was also the most abundant species in both the forest and the village.

## Conclusion

Vector control tools need to be adapted to the local context taking into account human and vector behaviour. A thorough knowledge of the biology, ecology and behaviour of vector species is essential to understand malaria transmission and to design appropriate vector control strategies. In Southeast Asia malaria is becoming more and more restricted to specific areas where human and vector characteristics alter the known features of malaria epidemiology, requiring alternative or adapted control strategies. This study showed clearly that risk of malaria transmission in the malaria foci is spread over the entire night, from dusk to dawn, requiring a combination of complementary vector control measures, such as LLIH and LN that can be used during different periods of the night. Moreover, with the progressive elimination of malaria in Southeast Asia, it can be expected that *P. vivax *becomes more prominent. Transmission of this *Plasmodium *species is not only assured by the main vectors but also by less known vectors as shown in this study. This poses new challenges when changing objectives from control to elimination of malaria from Southeast Asia and the need to focus not only on the so-called main vector species.

## Competing interests

The authors declare that they have no competing interests

## Authors' contributions

WVB, MC, and HDT designed the study. WVB supervised the work critically at all stages, carried out the data analysis and drafted the manuscript. HDT, LXH, NVH, NVC and NDL facilitated and carried out the field work. LD, PR carried out the ELISA assays and molecular identification of the collected mosquitoes and critically reviewed the manuscript. NS contributed to the statistical analysis and critically reviewed the manuscript. MC and UD critically reviewed the manuscript. All authors read and approved the final manuscript.
